# Design, Synthesis, and Biological Evaluation of Benzylamino-Methanone Based Cholesteryl Ester Transfer Protein Inhibitors

**DOI:** 10.3390/molecules15085721

**Published:** 2010-08-19

**Authors:** Ghassan Abu Sheikha, Reema Abu Khalaf, Areej Melhem, Ghadeer Albadawi

**Affiliations:** Department of Pharmaceutical Sciences, Faculty of Pharmacy, Al-Zaytoonah Private University of Jordan, Amman, Jordan; E-mail: pharmacy@alzaytoonah.edu.jo

**Keywords:** CETP inhibitors, high-density lipoprotein, pharmacophore modeling, quantitative structure-activity relationship, benzylamino-methanones

## Abstract

Cholesteryl ester transfer protein (CETP) is a glycoprotein involved in transporting lipoprotein particles and neutral lipids between high-density lipoprotein (HDL) and low density lipoproteins (LDL) and therefore its a proper target for treating dyslipidemia and related disorders. Guided by our previosuly-reported pharmacophore and QSAR models for CETP inhibition, we synthesized and bioassayed a series of benzylamino-methanones. The most potent illustrated 30% CETP inhibition at 10 μM.

## Introduction

Atherosclerosis is the main cause for arterial dysfunction that limits blood flow to vessels in the peripheral vasculature and is finally marked as coronary artery disease (CAD) [1]. A number of epidemiological studies have established an inverse relationship between serum high-density lipoprotein (HDL) cholesterol levels and the incidence of ischemic heart disease [2]. HDL removes excess cholesterol from peripheral tissues to the liver for biliary elimination [3]. CETP, a 476-residue glycoprotein, is engaged in interchanging lipoprotein particles and neutral lipids, including cholesteryl esters, phospholipids and triglycerides between HDL and low density lipoproteins (LDL). CETP, as revealed by X-ray crystallography (PDB code: 2OBD, resolution 2.2 Å), has a huge highly lipophilic binding site capable of binding up to four lipid molecules [4]. In human plasma, CETP plays a probably proatherogenic task by moving cholesteryl esters from HDL to very-low density lipoprotein (VLDL) and LDL particles, thereby lowering atheroprotective HDL cholesterol and raising proatherogenic VLDL and LDL cholesterols. Obviously, the risk of CAD is proportional to the plasma levels of CETP [5]. Actually, It is rather frequent within the CAD population to have elevated CETP plasma protein levels that are 2- to 3-fold higher than concentrations typically found in the plasma of normal subjects (1–3 µg/mL) [6].

Indication exists that the outcomes of CETP activity may depend on the metabolic setting, particularly on triglyceride levels. Therefore, pharmacological CETP inhibition may reduce the risk of CAD in humans, but only in those with high triglyceride levels [5].

**Figure 1:**
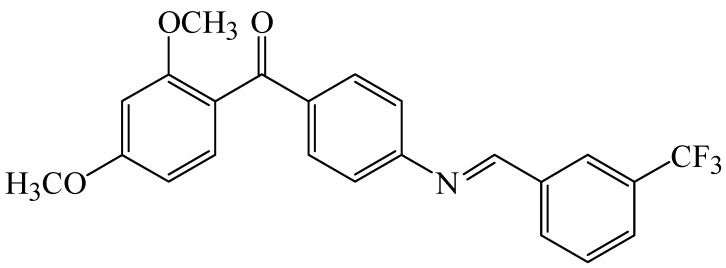
The structure of benzylidene-amino methanone derivative **1**.

The inaccessibility of a reasonable high resolution crystallographic structure for CETP combined with its large binding pocket locked up most modeling-related discovery projects to ligand-based approaches particularly quantitative structure-activity relationship analysis (QSAR) [7,8,9,10,11]. Earlier, we have developed ligand-based three-dimensional (3D) pharmacophores integrated within a self-consistent QSAR model for CETP inhibitors. The pharmacophore models were used as 3D search queries to mine 3D libraries for new CETP inhibitors, while the QSAR model predicted their biological activities and therefore prioritize them for *in vitro* evaluation [12]. Additionally, we recently described the synthesis of several benzylidene-amino methanones, (including **1**, Figure 1), as representatives of a new series of simple CETP inhibitors [12]. Herein we describe our efforts to optimize the activity of this series of compounds through reduction of the imine double bond to the corresponding amine analogues (compounds **19**–**30**, Scheme 2).

**Scheme 1:**
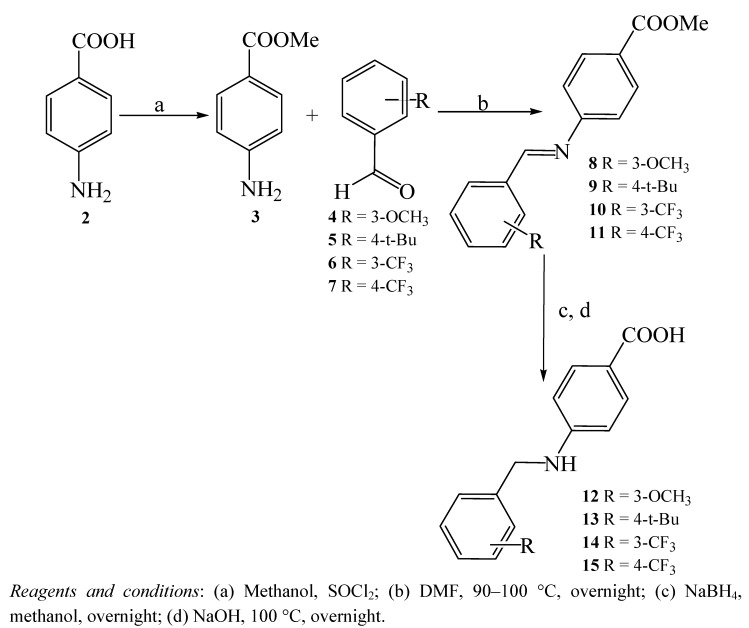
Synthesis of 4-aminobenzoic acid derivatives **12**–**15**.

The new compounds are expected to have better anti-CETP bioactivities compared to the previously synthesized rigid benzylidene-amino methanones due to the enhanced flexibility of the amino analogues, which should allow better fit values against the pharmacophores *i.e.* to fit both QSAR-emerged pharmacophores instead of mapping just one of them. Furthermore, amino-derivatives are more stable than imines in aqueous conditions [13]. On the other hand, reduction of imines into amines can alter their physicochemical properties such as lipophilicity and basicity of the nitrogen, whereby the amines are more water soluble and more basic than their imine analogues. This property modification can influence the anti-CETP activity of the synthesized compounds. The synthesis commenced by preparing different substituted imine intermediates **8**–**11 **(Scheme 1). Imines are typically formed by reversible acid-catalyzed condensation of amines and aldehydes with extrusion of water through either azeotropic distillation or by employing chemical drying agents [14].

**Scheme 2:**
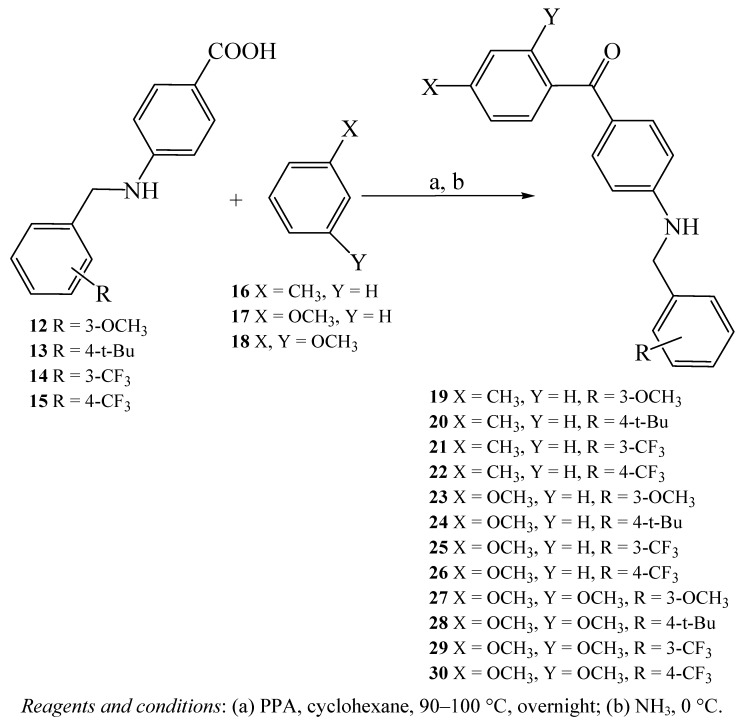
Synthesis of benzylamino-methanones **19**–**30**.

Subsequently, the imine intermediates were reduced to their corresponding amines **12**–**15 **(Scheme 1) which were then used to prepare the final benzylamino-methanones, in modest to reasonable yields, *via* Friedel–Crafts acylation of the substituted benzene derivatives **16**–**18** in the presence of polyphosphoric acid (PPA), as reported in Scheme 2 [15]. The structures proposed for compounds **19**–**30 **were confirmed via elemental analyses, IR and ^1^H- and ^13^C-NMR spectroscopy (see the Experimental section).

## Results and Discussion

**Figure 2:**
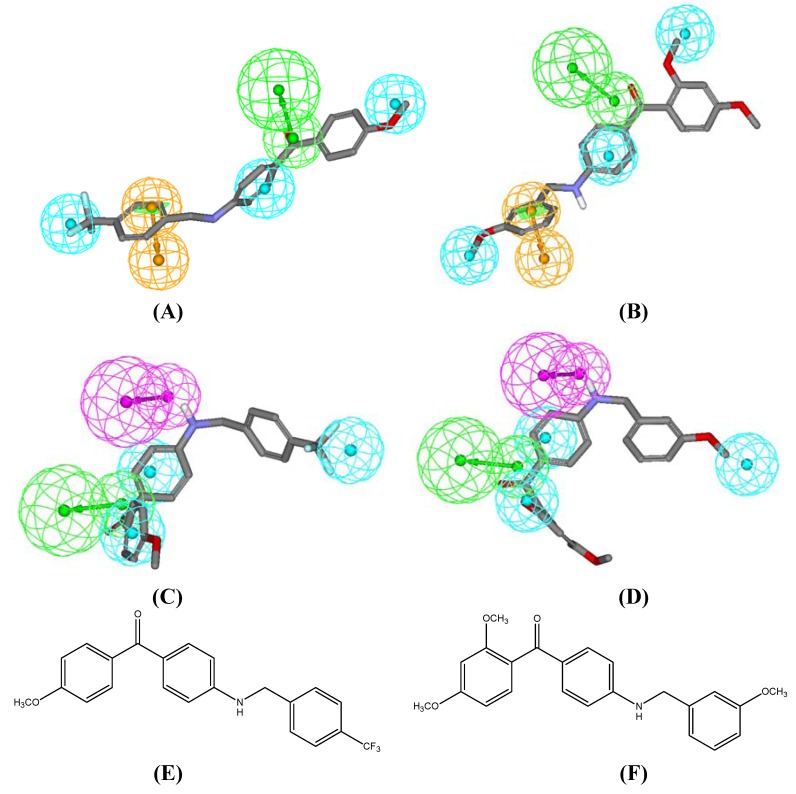
The binding pharmacophore hypotheses emerged in the optimal QSAR model (Hydrogen bond acceptor as green vectored spheres, hydrophobic features as blue spheres, ring aromatic as orange vectored spheres, Hydrogen bond donor as violet vectored spheres): (A) Hypo4/8 mapping **26**, (B) Hypo4/8 mapping **27**, (C) Hypo12/4 mapping **26**, (D) Hypo12/4 mapping **27**, (E) and (F) The chemical structures of **26** and **27**, respectively.

In the current work, the imine intermediates were prepared from reaction of trifluoro-*m*-tolualdehyde, trifluoro-*p*-tolualdehyde, 3-methoxybenzaldehyde and 4-tert-butylbenzaldehyde with the methyl ester of 4- aminobenzoic acid (**3**) as illustrated in Scheme 1 [16]. The best yield was obtained when **3**, dissolved in DMF, reacted with trifluoro-*p*-tolualdehyde to yield **15** (92.2%). Afterward, the imine intermediates were reduced to their corresponding amines, which were then used to prepare the final benzylamino-methanones. The best yield was obtained upon reacting 4-aminobenzoic acid derivative **15** with toluene to yield **22** (96%). Scheme 2 shows the new benzylamino-methanone derivatives **19**–**30**, while Figure 2 shows how Hypo4/8 and Hypo12/4 map compounds **26 **and **27**.

As can be noticed from Table 1, the benzylamino-methanone compounds have enhanced flexibility reflected by their fit values against Hypo12/4 in comparison to their benzylidene-amino methanones which do not fit Hypo12/4.

In this work, we intended to make the compounds more flexible in order to enhance their pharmacophore mapping in an attempt to increase their anti-CETP bioactivities, but it seems that the entropic cost contribution overcomes the enhanced flexibility contribution to the bioactivity. Therefore, pharmacophore fitness is just one of many factors that can influence the activity.

The results of anti-CETP activity tests, presented in Table 1, demonstrate that compound **26 **exhibited appreciable activity against CETP. Although our newly synthesized benzylamino-methanones are of lower potency than some published CETP inhibitors, these derivatives are characterized by their novel scaffold that could be a promising lead for further optimization.

The new compounds **19**–**30** were synthesized to explore how the CETP inhibitory activity is affected by the expansion of the structure, *i.e. meta *or *para *substitution, and the electronic properties of the substituent, *i.e.* electron donating or with-drawing group.

**Table 1:** The synthesized benzylamino-methanones with their fit values, corresponding QSAR estimates and *in vitro* bioactivities.

Compound	Fit values against		QSAR-based estimates		*In vitro* % inhibition of CETP at 10 μM	*In vitro* IC_50 _(μM)
	Hypo4/8	Hypo12/4	Log(1/IC_50_)	IC_50_(μM)		
**19**	7.9	2.2	-0.936	8.6	16.4 ± 3.0	—
**20**	8.3	0.8	-1.25	17.8	21.6 ± 0.8	60.3 (0.99)^a^
**21**	6.5	1	-1.282	19.1	16.7 ± 0.6	—
**22**	7.6	1.3	-0.86	7.2	12.6 ± 2.6	—
**23**	8.6	2	-1.111	12.9	20.0 ± 3.2	66.1 (0.99)^a^
**24**	8.3	1.8	-1.289	19.5	23.3 ± 2.3	51.3 (1.00)^a^
**25**	8.2	0.3	-1.051	11.2	9.8 ± 0.4	—
**26**	7.8	2.3	-0.754	5.7	29.9 ± 2.8	25.1 (0.99)^a^
**27**	9.4	3.1	-0.954	9	24.3 ± 2.7	36.3 (0.99)^a^
**28**	9.4	2.8	-0.823	6.7	21.3 ± 2.5	61.7 (0.99)^a^
**29**	8.5	1.4	-1.314	20.6	19.5 ± 0.6	—
**30**	8.8	2.1	-1.089	12.3	19.8 ± 2.9	—

^a^ This value represents the correlation coefficient of the corresponding dose-response line at three concentrations.

As a general trend, the inhibitory activity of compound **26**, that is *para*-substituted (R) with a trifluoromethyl electron withdrawing group and fully extended, decreases with the trifluoromethyl group on the *meta* position as in compound **25**. Furthermore, *meta* and *para* substitution (R) with electron donating groups such as *t*-Bu in compounds **20** and **24**, and methoxy in compound **23 **seems to conserve the anti-CETP activity. Moreover, mono- (X) or di-substitution (X and Y) of the aromatic ring, as in compounds **24** and **28**, respectively, appears not to have a considerable effect on the inhibitory activity.

Compounds **19**–**30** were tested against CETP at 10 μM concentrations and exhibited anti-CETP activity ranging from 9.8 to 29.9 %. Compound **26** displayed the best activity as reported in Table 1. Furthermore, *in vitro* IC_50_ values were determined for the most active compounds and approximately 3 to 9 fold differences were observed between QSAR-based IC_50_ estimates and the experimental IC_50 _values.

## Conclusions

In conclusion, we have successfully achieved synthetic exploration of a new series of aromatic amines as CETP inhibitors. They showed comparable activities to their benzylidene-amino methanones analogues [12] which reveals that flexibility of these amines was not enough to influence their antiCETP activity. We are currently in the process of preparing new compounds of better bioactivity profiles.

## Experimental

### General methods

Melting points were measured using Gallenkampf melting point apparatus and are uncorrected.^1^H- NMR and ^13^C-NMR spectra were collected on a Varian Oxford NMR^300^ spectrometer. The samples were dissolved in CDCl_3_. Mass spectrometry was performed using LC Mass Bruker Apex-IV mass spectrometer utilizing an electrospray interface. Infrared spectra were recorded using Shimadzu IRAffinity-1 spectrophotometer. The samples were dissolved in CHCl_3_and analysed as thin solid films using NaCl plates. Analytical thin layer chromatography (TLC) was carried out using pre-coated aluminum plates and visualized by UV light (at 254 and/ or 360 nm). Elemental analysis was performed using EuroVector elemental analyzer. Chemicals and solvents were purchased from the corresponding companies (Sigma-Aldrich, Riedel-de Haen, Fluka, BDH Laboratory Supplies and Promega Corporation) and were used in the experimentation without further purification.

### General procedure for the synthesis of 4-aminobenzoic acid derivatives 12–15

4-Aminobenzoic acid (**2**, 1.37 g, 10 mmol) was dissolved in methanol (100 mL), and then thionyl chloride (200 mmol) was added at 0 °C. The mixture was stirred at room temperature for 20–30 minutes, followed by refluxing at 65–70 °C overnight. Evaporation of the solvent was carried out, followed by neutralization using K_2_CO_3_ and extraction with CH_2_Cl_2_ (3 × 20 mL). The combined extracts were dried on anhydrous Na_2_SO_4_ and filtrated to give 4-amino-benzoic acid methyl ester (**3**,96%). Subsequently, **3** (1.52 g, 10 mmol) was disolved in DMF (10 mL), then an aldehyde (**4**–**7**, 25 mmol) was added. The mixture was heated between 100–150 °C overnight. After removing DMF, methanol (15 mL) was added to the reaction mixture, followed by gradual addition of NaBH_4_ (4 equivalents) and stirring at room temperature overnight. The residue, after evaporation of the solvent, was purified by column chromatography eluting with cyclohexane/EtOAc (90:10). Next, desterification was carried out by refluxing with 1M NaOH (2.6 equivalents) at 100 °C overnight. Then, the reaction mixture was neutralized with HCl and extracted with CHCl_3_ (3 × 20 mL). The combined extracts were dried on anhydrous Na_2_SO_4_ and filtered. 

*4-(3-Methoxybenzylamino)-benzoic acid* (**12**)*.* Evaporation of the solvent gave **12 **as an off-white powder (88%); mp. 160–161 °C; ^1^H-NMR (300 MHz, CDCl_3_) *δ* 3.68 (s, 3H), 4.26 (s, 2H), 4.72 (br s, 1H), 6.55 (d, *J* = 8.8 Hz, 2H), 6.77 (dd, *J* = 8.1, 1.4 Hz, 1H), 6.86–6.89 (m, 2H), 7.21 (t, *J* = 8.1 Hz, 1H), 7.61 (d, *J* = 8.8 Hz, 2H), 12.03 (br s, 1H); ^13^C-NMR (300 MHz, CDCl_3_) *δ* 46.3 (1C), 55.4 (1C), 111.7 (1C), 112.5 (1C), 113.3 (1C), 117.6 (1C), 119.8 (1C), 130.0 (2C), 131.6 (2C), 141.7 (1C), 152.9 (1C), 159.9 (1C), 168.0 (1C); IR (thin film) cm^-1^ 3437, 3059, 2940, 1655, 1597, 1489, 1427, 1285, 1177.

*4-(4-tert-Butylbenzylamino)-benzoic acid *(**13**). Evaporation of the solvent gave **13 **as an off-white powder (83%); mp. 210–211 °C; ^1^H-NMR (300 MHz, CDCl_3_) *δ* 1.27 (s, 9H), 4.36 (s, 2H), 4.80 (br s, 1H), 6.60 (d, *J* = 8.7 Hz, 2H), 7.26–7.36 (m, 4H), 7.94 (d, *J* = 8.7 Hz, 2H), 11.87 (br s, 1H); ^13^C-NMR (300 MHz, CDCl_3_) *δ* 31.4 (3C), 34.6 (1C), 47.4 (1C), 111.6 (2C), 117.6 (1C), 125.8 (2C), 127.4 (2C), 132.4 (2C), 135.2 (1C), 150.7 (1C), 152.5 (1C), 172.4 (1C); IR (thin film) cm^-1^ 3426, 3017, 2963, 1670, 1605, 1524, 1478, 1420, 1292.

*4-(3-Trifluoromethylbenzylamino)-benzoic acid *(**14**). Evaporation of the solvent gave **14 **as an off-white powder (77%); mp. 168–169 °C; ^1^H-NMR (300 MHz, CDCl_3_) *δ* 4.41 (s, 2H), 4.77 (br s, 1H), 6.57 (d, *J* = 8.6 Hz, 2H), 7.53–7.66 (m, 6H), 11.93 (br s, 1H); ^13^C-NMR (300 MHz, CDCl_3_) *δ* 45.7 (1C), 111.7 (2C), 118.0 (1C), 124.0 (1C), 124.1 (1C), 127.6 (1C), 129.9 (1C), 131.6 (2C), 131.8 (2C), 141.7 (1C), 152.6 (1C), 167.9 (1C); IR (thin film) cm^-1^ 3456, 3062, 2920, 1674, 1605, 1516, 1481, 1424, 1316, 1107.

*4-(4-Trifluoromethylbenzylamino)-benzoic acid* (**15**). Evaporation of the solvent gave **15 **as a pale yellow powder (93%);mp. 189–190 °C; ^1^H-NMR (300 MHz, CDCl_3_) *δ* 4.41 (s, 2H), 4.79 (br s, 1H), 6.54 (d, *J* = 8.8 Hz, 2H), 7.51 (d, *J* = 8.1 Hz, 2H), 7.59–7.67 (m, 4H), 12.02 (br s, 1H); ^13^C-NMR (300 MHz, CDCl_3_) *δ* 45.8 (1C), 111.7 (2C), 118.0 (1C), 123.1 (1C), 125.8 (2C), 127.8 (1C), 128.2 (2C), 131.6 (2C), 145.2 (1C), 152.6 (1C), 167.9 (1C); IR (thin film) cm^-1 ^3414, 3042, 2920, 1659, 1605, 1520, 1462, 1424, 1323, 1122.

### General procedure for the synthesis of benzylamino-methanones 19–30

4-Aminobenzoic acid derivative **12**–**15** (2 mmol) was dissolved in cyclohexane (10 mL), and polyphosphoric acid (6.5 g) was added. Then a benzene derivative **16**–**18** (20 mmol) was added. The mixture was stirred carefully at 90–110 °C overnight and then poured on crushed ice. The solution was carefully made alkaline with 25% ammonia and then extracted with CHCl_3_ (3 × 20 mL). The combined extracts were dried on anhydrous Na_2_SO_4_ and filtered.

*[4-(3-Methoxybenzylamino)phenyl]-p-tolyl-methanone (**19**).* The residue, after evaporation of the solvent, was purified by column chromatography eluting with cyclohexane/EtOAc (80:20) to give pure **19 **as a yellow powder (22%); *R_f_* = 0.52 (CHCl_3_-MeOH, 98:2); mp. 123–124 °C; ^1^H-NMR (300 MHz, CDCl_3_) *δ* 2.32 (s, 3H), 3.78 (s, 3H), 4.37 (s, 2H), 4.65 (br s, 1H), 6.60 (d, *J* = 7.7 Hz, 2H), 6.80–6.94 (m, 2H), 7.22–7.28 (m, 4H), 7.62 (d, *J* = 7.2 Hz, 2H), 7.71 (d, *J* = 7.7 Hz, 2H); ^13^C-NMR (300 MHz, CDCl_3_) *δ* 21.6 (1C), 47.7 (1C), 55.3 (1C), 111.6 (2C), 112.8 (1C), 113.1 (1C), 119.6 (1C), 126.9 (1C), 127.7 (2C), 129.9 (2C), 130.6 (1C), 132.9 (2C), 136.2 (1C), 140.0 (1C), 141.9 (1C), 151.7 (1C), 160.0 (1C), 195.1 (1C); IR (thin film) cm^-1^ 3356, 3017, 2963, 1636, 1593, 1528, 1468, 1262, 1150; MS (ESI, positive mode) *m/z* [*M*+H]^+^ 332.16451 (C_22_H_22_NO_2_ requires 332.15723); Anal. Calcd for C_22_H_21_NO_2_: C 79.73, H 6.39, N 4.23, found: C 79.68, H 6.41, N 4.21.

*[4-(4-tert-Butylbenzylamino)phenyl]-p-tolyl-methanone (**20**).* The residue, after evaporation of the solvent, was purified by column chromatography eluting with cyclohexane/EtOAc (85:15) to give pure **20 **as a yellow powder (23%); *R_f_* = 0.57 (CHCl_3_-MeOH, 98:2); mp. 140–141 °C; ^1^H-NMR (300 MHz, CDCl_3_) *δ* 1.23 (s, 9H), 2.32 (s, 3H), 3.83 (s, 2H), 4.12 (br s, 1H), 6.65 (d, *J* = 8.6 Hz, 2H), 7.10 (d, *J* = 7.8 Hz, 2H), 7.16–7.30 (m, 4H), 7.62–7.75 (m, 4H); ^13^C-NMR (300 MHz, CDCl_3_) *δ* 22.8 (1C), 29.5 (3C), 32.0 (1C), 39.1 (1C), 121.1 (1C), 123.7 (1C), 126.8 (1C), 128.3 (2C), 128.9 (2C), 129.0 (2C), 129.4 (2C), 130.2 (2C), 130.8 (1C), 132.9 (2C), 143.0 (1C), 150.7 (1C), 194.2 (1C); IR (thin film) cm^-1^ 3422, 3337, 3024, 2963, 1636, 1586, 1501, 1439, 1319; MS (ESI, positive mode) *m/z* [*M*+H]^+^ 358.21016 (C_25_H_28_NO requires 358.20926); Anal. Calcd for C_25_H_27_NO: C 83.99, H 7.61, N 3.92, found: C 83.87, H 7.58, N 3.97.

*p-Tolyl-[4-(3-trifluoromethylbenzylamino)phenyl]-methanone *(**21**). The residue, after evaporation of the solvent, was purified by column chromatography eluting with cyclohexane/EtOAc (80:20) to give pure **21** as a reddish-orange oil (39%); *R_f_* = 0.70 (CHCl_3_-MeOH, 98:2);^ 1^H-NMR (300 MHz, CDCl_3_) *δ* 2.38 (s, 3H), 4.46 (s, 2H), 4.78 (br s, 1H), 6.58 (d, *J* = 5.2 Hz, 2H), 7.23 (d, *J* = 9.2 Hz, 2H), 7.43–7.74 (m, 8H); ^13^C-NMR (300 MHz, CDCl_3_) *δ* 21.5 (1C), 47.3 (1C), 111.6 (2C), 111.8 (1C), 113.7 (1C), 125.1 (1C), 127.6 (1C), 128.6 (2C), 129.2 (2C), 129.8 (1C), 132.9 (2C), 134.3 (1C), 136.2 (1C), 138.8 (1C), 141.9 (1C), 143.5 (1C), 151.4 (1C), 195.1 (1C); IR (thin film) cm^-1 ^3356, 3024, 2924, 1651, 1593, 1527, 1439; MS (ESI, positive mode) *m/z* [*M*+H]^+^ 370.14133 (C_22_H_19_F_3_NO requires 370.13405).

*p-Tolyl-[4-(4-trifluoromethylbenzylamino)phenyl]-methanone* (**22**). The residue, after evaporation of the solvent, was purified by column chromatography eluting with cyclohexane/EtOAc (80:20) to give pure **22 **as a dark-yellow powder (96%); *R_f_* =0.49 (CHCl_3_-MeOH, 98:2); mp. 115–116 °C;^ 1^H-NMR (300 MHz, CDCl_3_) *δ* 2.35 (s, 3H), 4.49 (s, 2H), 4.82 (br s, 1H), 6.60 (dd, *J* = 9.0, 1.9 Hz, 2H), 7.27 (t, *J* = 8.7 Hz, 2H), 7.42 (d, *J* = 6.2 Hz, 2H), 7.60 (dd, *J* = 9.0, 1.9 Hz, 2H), 7.66–7.78 (m, 4H); ^13^C-NMR (300 MHz, CDCl_3_) *δ* 21.6 (1C), 47.3 (1C), 111.7 (2C), 127.0 (1C), 127.7 (1C), 128.8 (2C), 129.4 (2C), 130.2 (2C), 130.5 (2C), 130.6 (1C), 130.7 (2C), 131.1 (1C), 132.9 (1C), 142.0 (1C), 152.0 (1C), 195.1 (1C); IR (thin film) cm^-1 ^3356, 3021, 2920, 1647, 1597, 1528, 1451; MS (ESI, positive mode) *m/z* [*M*+H]^+^ 370.14133 (C_22_H_19_F_3_NO requires 370.13405); Anal. Calcd for C_22_H_18_F_3_NO: C 71.53, H 4.91, N 3.79, found: C 71.48, H 4.95, N 3.67.

*[4-(3-Methoxybenzylamino)phenyl]-(4-methoxyphenyl)-methanone *(**23**).The residue, after evaporation of the solvent, was purified by column chromatography eluting with CHCl_3_/MeOH (99:1) to give pure **23 **as an orange oil (28%); *R_f_* = 0.44 (CHCl_3_-MeOH, 98:2);^ 1^H-NMR (300 MHz, CDCl_3_) *δ* 3.73 (s, 3H), 3.85 (s, 3H), 4.37 (s, 2H), 4.63 (br s, 1H), 6.59 (d, *J* = 8.7 Hz, 2H), 6.78–6.95 (m, 6H), 7.65–7.74 (m, 4H); ^13^C-NMR (300 MHz, CDCl_3_) *δ* 47.6 (1C), 55.3 (1C), 55.7 (1C), 111.3 (2C), 113.3 (2C), 120.3 (1C), 127.0 (1C), 129.0 (1C), 129.9 (1C), 130.8 (1C), 131.5 (1C), 132.0 (2C), 132.8 (2C), 140.1 (1C), 151.6 (1C), 160.0 (1C), 162.4 (1C), 194.3 (1C); IR (thin film) cm^-1^ 3352, 3005, 2925, 1636, 1597, 1528, 1458, 1316, 1258, 1169; MS (ESI, positive mode) *m/z* [*M*+H]^+^ 348.14725 (C_22_H_22_NO_3_ requires 348.15214).

*[4-(4-tert-Butylbenzylamino)phenyl]-(4-methoxyphenyl)-methanone *(**24**).The residue, after evaporation of the solvent, was purified by column chromatography eluting with cyclohexane/EtOAc (80:20) to give pure **24 **as a red oil (60%); *R_f_* = 0.52 (CHCl_3_-MeOH, 98:2);^ 1^H-NMR (300 MHz, CDCl_3_) *δ* 1.26 (s, 9H), 3.84 (s, 3H), 3.89 (s, 2H), 4.10 (br s, 1H), 6.65 (d, *J* = 8.2 Hz, 1H), 6.93 (d, *J* = 8.6 Hz, 2H), 7.11 (d, *J* = 8.2 Hz, 1H), 7.20 (d, *J* = 7.6 Hz, 2H), 7.29 (t, *J* = 7.6 Hz, 2H), 7.58–7.66 (m, 2H), 7.78 (d, *J* = 8.6 Hz, 2H); ^13^C-NMR (300 MHz, CDCl_3_) *δ* 31.4 (3C), 37.7 (1C), 38.2 (1C), 55.5 (1C), 123.7 (2C), 125.7 (1C), 126.7 (1C), 128.1 (1C), 128.4 (2C), 128.9 (2C), 131.1 (1C), 131.4 (2C), 133.9 (1C), 135.4 (1C), 138.6 (1C), 149.2 (1C), 149.6 (1C), 162.5 (1C), 194.5 (1C); IR (thin film) cm^-1 ^3476, 3364, 3005, 2963, 1620, 1601, 1508, 1458, 1420, 1254; MS (ESI, positive mode) *m/z* [*M*+H]^+^ 374.19873 (C_25_H_28_NO_2_ requires 374.20418).

*(4-Methoxy-phenyl)-[4-(3-trifluoromethyl-benzylamino)-phenyl]-methanone *(**25**). The residue, after evaporation of the solvent, was purified by column chromatography eluting with cyclohexane/EtOAc (80:20) to give pure **25 **as a red oil (33%); *R_f_* = 0.60 (CHCl_3_-MeOH, 98:2);^ 1^H-NMR (300 MHz, CDCl_3_) *δ* 3.82 (s, 3H), 4.46 (s, 2H), 4.73 (br s, 1H), 6.60 (dd, *J* = 6.9, 1.8 Hz, 2H), 6.93 (dd, *J* = 6.9, 1.8 Hz, 2H), 7.45–7.61 (m, 4H), 7.64–7.74 (m, 4H); ^13^C-NMR (300 MHz, CDCl_3_) *δ* 47.2 (1C), 55.5 (1C), 111.7 (2C), 113.4 (2C), 120.4 (1C), 123.9 (1C), 124.4 (1C), 127.5 (1C), 129.0 (1C), 129.3 (1C), 130.6 (1C), 130.9 (1C), 131.4 (2C), 132.0 (2C), 139.7 (1C), 151.2 (1C), 162.5 (1C), 194.3 (1C); IR (thin film) cm^-1^ 3348, 3032, 2932, 1636, 1601, 1528, 1455, 1327, 1169; MS (ESI, positive mode) *m/z* [*M*+H]^+^ 386.13624 (C_22_H_19_F_3_NO_2_ requires 386.12896).

*(4-Methoxyphenyl)-[4-(4-trifluoromethylbenzylamino)phenyl]-methanone* (**26**). The residue, after evaporation of the solvent, was purified by column chromatography eluting with CH_2_Cl_2_/EtOH (98:2) to give pure **26 **as a yellow oil (22%); *R_f_* = 0.56 (CHCl_3_-MeOH, 98:2); ^1^H-NMR (300 MHz, CDCl_3_) *δ* 3.71 (s, 3H), 4.41 (s, 2H), 4.70 (br s, 1H), 6.50 (dd, *J* = 9.5, 4.0 Hz, 2H), 6.89 (dd, *J* = 9.3, 3.2 Hz, 2H), 7.20 (t, *J* = 5.0 Hz, 2H), 7.38 (dd, *J* = 9.3, 3.2 Hz, 2H), 7.52 (t, *J* = 5.0 Hz, 2H), 7.64 (dd, *J* = 9.5, 4.0 Hz, 2H); ^13^C-NMR (300 MHz, CDCl_3_) *δ* 47.4 (1C), 55.9 (1C), 111.6 (1C), 111.9 (2C), 113.6 (2C), 122.5 (1C), 126.0 (2C), 127.7 (2C), 129.2 (1C), 131.5 (2C), 132.9 (2C), 142.8 (1C), 151.3 (1C), 157.1 (1C), 162.7 (1C), 194.4 (1C); IR (thin film) cm^-1^ 3345, 3035, 2963, 1636, 1597, 1531, 1462, 1323, 1165; MS (ESI, positive mode) *m/z* [*M*+H]^+^ 386.13624 (C_22_H_19_F_3_NO_2_ requires 386.12896).

*(2,4-Dimethoxyphenyl)-[4-(3-methoxybenzylamino)phenyl]-methanone *(**27**).The residue, after evaporation of the solvent, was purified by column chromatography eluting with cyclohexane/EtOAc (75:25) to give **27 **pure as a pink powder (53%); *R_f_* = 0.33 (CHCl_3_-MeOH, 98:2); mp. 126–127 °C; ^1^H-NMR (300 MHz, CDCl_3_) *δ* 3.68 (s, 3H), 3.77 (s, 3H), 3.83 (s, 3H), 4.34 (s, 2H), 4.66 (br s, 1H), 6.48–6.55 (m, 4H), 6.78–6.91 (m, 4H), 7.25 (t, *J* = 7.7 Hz, 1H), 7.99 (d, *J* = 8.7 Hz, 2H); ^13^C-NMR (300 MHz, CDCl_3_) *δ* 47.6 (1C), 55.3 (1C), 55.5 (1C), 55.7 (1C), 98.6 (1C), 104.2 (1C), 111.5 (2C), 112.8 (1C), 113.1 (1C), 119.6 (1C), 122.6 (1C), 127.7 (1C), 129.9 (1C), 131.1 (1C), 132.7 (2C), 140.1 (1C), 151.9 (1C), 158.8 (1C), 160.0 (1C), 162.3 (1C), 194.0 (1C); IR (thin film) cm^-1 ^3348, 3005, 2936, 1636, 1597, 1489, 1458, 1316, 1262, 1161; MS (ESI, positive mode) *m/z* [*M*+H]^+^ 378.15691 (C_23_H_24_NO_4_ requires 378.16271); Anal. Calcd for C_23_H_23_NO_4_: C 73.19, H 6.14, N 3.71, found: C 73.25, H 6.21, N 3.68.

*[4-(4-tert-Butylbenzylamino)phenyl]-(2,4-dimethoxyphenyl)-methanone *(**28**). The residue, after evaporation of the solvent, was purified by column chromatography eluting with cyclohexane/EtOAc (80:20) to give pure **28 **as a brown powder (82%); *R_f_* = 0.45 (CHCl_3_-MeOH, 98:2); mp. 138–139 °C; ^1^H-NMR (300 MHz, CDCl_3_) *δ* 1.27 (s, 9H), 3.70 (s, 3H), 3.77 (s, 3H), 3.89 (s, 2H), 4.08 (br s, 1H), 6.50 (d, *J* = 8.6 Hz, 2H), 6.58 (d, *J* = 8.3 Hz, 2H), 7.08 (d, *J* = 8.6 Hz, 2H), 7.24–7.31 (m, 3H), 7.57 (d, *J* = 8.3 Hz, 1H), 7.64 (d, *J* = 5.5 Hz, 1H); ^13^C-NMR (300 MHz, CDCl_3_) *δ* 31.4 (3C), 34.5 (1C), 37.6 (1C), 55.5 (1C), 55.6 (1C), 98.8 (1C), 104.5 (1C), 114.5 (1C), 122.5 (1C), 125.7 (2C), 128.0 (2C), 128.8 (1C), 131.1 (1C), 131.3 (2C), 133.8 (2C), 135.5 (1C), 149.6 (1C), 158.9 (1C), 162.5 (1C), 194.4 (1C); IR (thin film) cm^-1^ 3480, 3364, 3005, 2963, 1623, 1602, 1505, 1462, 1410, 1312, 1277, 1211; MS (ESI, positive mode) *m/z* [*M*+H]^+^ 404.22034 (C_26_H_30_NO_3_ requires 404.21474); Anal. Calcd for C_26_H_29_NO_3_: C 77.39, H 7.24, N 3.47, found: C 77.42, H 7.30, N 3.51. 

*(2,4-Dimethoxyphenyl)-[4-(3-trifluoromethylbenzylamino)phenyl]-methanone *(**29**).The residue, after evaporation of the solvent, was purified by column chromatography eluting with cyclohexane/EtOAc (75:25) to give pure **29 **as an olive-green oil (32%); *R_f_* = 0.58 (CHCl_3_-MeOH, 98:2); ^1^H-NMR (300 MHz, CDCl_3_)*δ* 3.71 (s, 3H), 3.82 (s, 3H), 4.43 (s, 2H), 4.83 (br s, 1H), 6.46–6.55 (m, 4H), 7.25 (dd, *J* = 7.1, 2.0 Hz, 1H), 7.42–7.58 (m, 4H), 7.67 (d, *J* = 8.8 Hz, 2H); ^13^C-NMR (300 MHz, CDCl_3_) *δ* 47.2 (1C), 55.5 (1C), 55.7 (1C), 98.9 (1C), 104.2 (1C), 111.6 (2C), 122.5 (1C), 123.9 (1C), 124.0 (1C), 124.3 (1C), 124.4 (1C), 128.1 (1C), 129.3 (1C), 130.6 (1C), 131.1 (1C), 132.6 (2C), 139.7 (1C), 151.6 (1C), 158.9 (1C), 162.4 (1C), 194.1 (1C); IR (thin film) cm^-1^ 3337, 3009, 2936, 1636, 1597, 1528, 1505, 1458, 1327, 1161; MS (ESI, positive mode) *m/z* [*M*+H]^+^ 416.14680 (C_23_H_21_F_3_NO_3_ requires 416.13953).

*(2,4-Dimethoxyphenyl)-[4-(4-trifluoromethylbenzylamino)phenyl]-methanone* (**30**).The residue, after evaporation of the solvent, was purified by column chromatography eluting with cyclohexane/EtOAc (75:25) to give pure **30 **as a dark-brown oil (50%); *R_f_* = 0.44 (CHCl_3_-MeOH, 98:2); ^1^H-NMR (300 MHz, CDCl_3_)*δ* 3.72 (s, 3H), 3.82 (s, 3H), 4.45 (s, 2H), 4.76 (br s, 1H), 6.48 (dd, *J* = 7.9, 1.9 Hz, 2H), 6.53 (d, *J* = 8.0 Hz, 2H), 7.26 (d, *J* = 2.9 Hz, 1H), 7.45 (d, *J* = 8.1 Hz, 2H), 7.57 (d, *J* = 8.1 Hz, 2H), 7.66 (dd, *J* = 7.9, 1.9 Hz, 2H); ^13^C-NMR (300 MHz, CDCl_3_) *δ* 47.1 (1C), 55.5 (1C), 55.7 (1C), 98.5 (1C), 98.9 (1C), 104.4 (1C), 111.6 (2C), 122.5 (1C), 125.6 (1C), 125.8 (2C), 127.4 (2C), 128.2 (1C), 129.6 (1C), 132.6 (2C), 142.7 (1C), 151.5 (1C), 158.9 (1C), 162.4 (1C), 194.0 (1C); IR (thin film) cm^-1 ^3345, 3009, 2940, 1636, 1597, 1528, 1505, 1462, 1323, 1161; MS (ESI, positive mode) *m/z* [*M*+H]^+^ 416.14680 (C_23_H_21_F_3_NO_3_ requires 416.13953).

### CETP inhibition assay

CETP inhibitory bioactivities were assayed by fluorescent-CE transfer employing commercially available kit (BioVision, Mountain View, CA, USA). The assay kit is based on donor molecule containing fluorescent self-quenched neutral lipid that is transferred to an acceptor molecule in the presence of CETP (from rabbit serum). CETP-mediated transfer of the fluorescent neutral lipid to the acceptor molecule results in increase in fluorescence. Inhibition of CETP will prevent lipid transfer and therefore decrease fluorescence intensity. 

The assay procedure can be described briefly as follows: an aliquot of rabbit serum (1.5 µL) was added to testing sample (160 µL). Then the master mix, provided in the assay kit (donor molecule, acceptor molecule and assay buffer, 20 µL) was added, mixed well, and the volume was completed to 203 µL with the provided assay buffer. After incubation at 37 °C for 1 hour, fluorescence intensity (Excitation λ: 465 nm; Emission λ: 535 nm) was read in a FLX800TBI Microplate Fluorimeter (BioTek Instruments, Winooski, VT, USA). 

The tested compounds were initially dissolved in DMSO to yield 10 mM stock solutions and subsequently diluted to the required concentrations using distilled deionized water. The final concentration of DMSO was adjusted to 0.1%. The percentage of residual activity of CETP was determined for each compound by comparing the activity of CETP in the presence and absence of the tested compound. Positive controls were tested to assess the degree of CETP inhibition by 0.1% DMSO. CETP was not affected by DMSO. Negative controls lacking rabbit serum were used as background. All measurements were conducted in duplicates.
